# The shark-tuna dichotomy: why tuna lay tiny eggs but sharks produce large offspring

**DOI:** 10.1098/rsos.180453

**Published:** 2018-08-15

**Authors:** Richard M. Sibly, Astrid Kodric-Brown, Susan M. Luna, James H. Brown

**Affiliations:** 1 School of Biological Sciences, University of Reading, Reading, UK; 2 Biology Department, University of New Mexico, Albuquerque, NM, USA; 3 FishBase Information and Research Group, Inc. (FIN), IRRI, Los Baños, Laguna, Philippines

**Keywords:** optimal offspring size, ESS, fish reproductive strategies, number–size trade-off, pearl survivorship curve

## Abstract

Teleosts such as tunas and billfish lay millions of tiny eggs weighing on the order of 0.001 g, whereas chondrichthyes such as sharks and rays produce a few eggs or live offspring weighing about 2% of adult body mass, as much as 10 000 g in some species. Why are the strategies so extreme, and why are intermediate ones absent? Building on previous work, we show quantitatively how offspring size reflects the relationship between growth and death rates. We construct fitness contours as functions of offspring size and number, and show how these can be derived from juvenile growth and survivorship curves. Convex contours, corresponding to Pearl Type 1 and 2 survivorship curves, select for extremes, either miniscule or large offspring; concave contours select for offspring of intermediate size. Of particular interest are what we call critical straight-line fitness contours, corresponding to log-linear Pearl Type 3 survivorship curves, which separate regimes that select for opposite optimal offspring sizes.

## 1. Introduction

**Figure 1.**
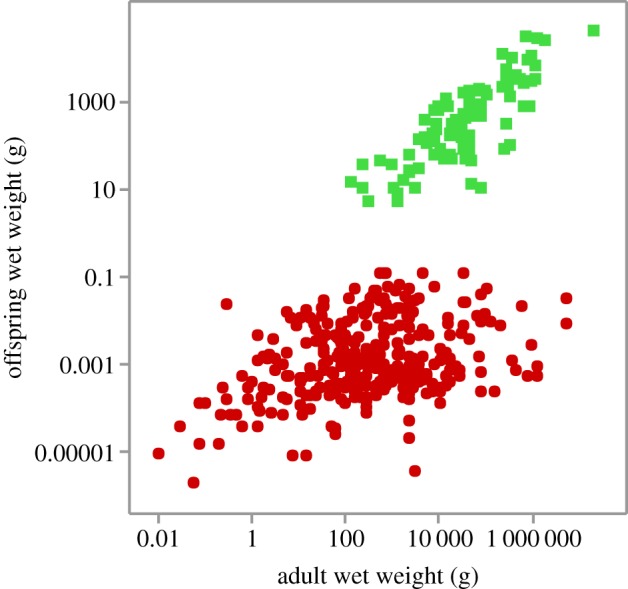
Body mass of newborn chondrichthyes (green squares) and teleosts (red circles) as a function of adult body mass. Data from FishBase compiled December 2016 [4] using conversions mass (g) = 0.01 length (cm)^3^ except egg mass (g) = 0.52 diameter (cm)^3^. OLS regression for chondrichthyes (±s.e.): log_10_ offspring wt = −0.94 ± 0.26 + 0.76 ± 0.06 log_10_ adult wt (*t*_96_ = 13.8, p≪0.001).

A fundamental trade-off in life history is how overall reproductive effort is allocated to produce either many small or a few large offspring (e.g. [1–3]). An especially dramatic example in animals is afforded by marine fish, which exhibit one of the two extreme reproductive strategies, while the size of offspring varies by at least 7 orders of magnitude (figure 1). The large teleosts (bony fishes that include tunas, billfish, marine sunfish and groupers) have external fertilization and produce enormous numbers of tiny eggs that hatch out as free-living larvae [5–8]. Teleost egg size averages about 1 mg and is only very weakly correlated with adult body size. The chondrichthyes (cartilaginous sharks, rays and chimeras) and the ‘living fossil’ coelacanth have internal fertilization and produce a relatively small number of relatively large offspring, either large, well-protected eggs or live-born young. The size of newborn chondrichthyes scales positively and is on average 2% adult body mass (range 0.01–15%, figure 1, [8–12]). Some large sharks give birth to live offspring weighing more than 10 000 g.

So, a challenging question is how have these two groups of marine fish, with such contrasting life histories, been able to occur together for about 200 Myr in many different marine habitats. This question has been the subject of repeated investigation [3,6,7,12–17]. Producing large, well-developed offspring can be viewed as a form of parental care, and hypotheses to explain the well-documented correlation between parental care and propagule size in animals have focused on how mortality rates of offspring vary with their size [16]. Computer simulations of foraging suggest that the many-small tuna strategy does best when prey are abundant and clumped [6]. This led to the suggestion that the teleost explosion was driven by the concurrent evolution of the copepods, which provided a rich food source for their numerous, tiny larvae [13]. Using simple assumptions from metabolic scaling theory about the size-dependence of growth and death rates [8,12] showed that ordinarily the tuna strategy is best, but if death rate becomes increasingly density-dependent with increasing juvenile size, this may give the advantage to a shark strategy, leading to the observed positive correlation between the sizes of offspring and adults.

Existing theory and data beg the question of why the shark-tuna dichotomy represents two alternative evolutionary stable strategies. The long history of coexistence of diverse chondrichthyes and teleosts suggests that the extreme few-large and many-small offspring strategies are approximately equally fit, whereas intermediate strategies are absent because they have been selected against. In this paper, we show how fitness contours can be obtained directly from juvenile growth and survivorship curves, and build upon earlier studies to obtain more general results showing how the shark and tuna strategies, with either a few, large or many small offspring, can confer equal fitness. We follow others in assuming the existence of an allocation trade-off such that the product of offspring size and number is fixed. When this assumption holds, we show that when the death rate of juveniles exceeds the growth rate, the shark strategy is fitter because more offspring survive to adulthood, but when the growth rate exceeds the death rate, the tuna strategy is superior because some, out of the enormous number of tiny offspring, grow fast enough to survive to adulthood.

## 2. How fitness contours select for initial offspring size

In this section, we characterize the optimal reproductive strategy in terms of offspring size and number. The action of selection is investigated by calculating the fitness consequences of reproductive options. Net reproductive rate, *R*_0_, is used as the underlying measure of fitness, and it is assumed that populations using the optimal strategy are in steady state at *R*_0_ = 1 [18,19]. We do not consider here how density dependence maintains the population at steady state, but assume implicitly that this is achieved by adjustments of the components of *R*_0_ (‘ecological compensation’ [20]). In simple iteroparous life histories in which fecundity *n* is the same each time an individual breeds, juvenile survivorship is *S_j_* and adult survivorship between reproductive events is *S*_a_; net reproductive rate is given by $\;R_{0}\;=\;nS_{j}(1+S_{a}+S_{a}^{2}+S_{a}^{3}+\cdots )$. In the analysis that follows we are only interested in life-history options from birth until the offspring reach a certain size *m*_L_, so we assume *S*_a_ and survivorship from *m*_L_ to adult size are both fixed and use the number of offspring that survive from each breeding event to size *m*_L_ as our operational fitness measure.

**Figure 2.**
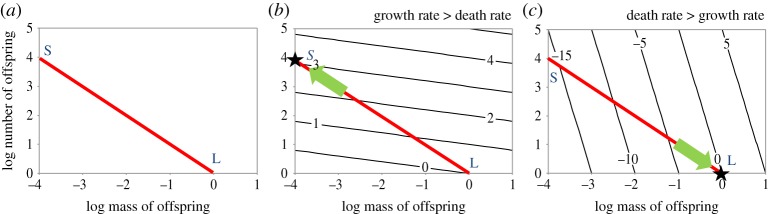
Reproductive options and fitness contours represented in log_*e*_–log_*e*_ plots of initial offspring number *y*_0_ versus initial offspring mass *x*_0_. (*a*) The reproductive options, represented by the red constraint line, lie on a straight line of slope –1. The smallest size strategy, S, is to produce the maximum possible number of tiny offspring; the largest size strategy, L, is to produce a single offspring of the largest possible size. (*b*) If growth rate exceeds the death rate, the optimal strategy is indicated by the black star labelled S. Reproductive options with fitness contours are represented by black lines, and the green arrow indicates the direction of selection increasing fitness subject to the physiological constraint. Fitness contours are drawn using equation (2.2) for a case in which growth rate exceeds death rate by a factor of five, so that *d*/*g* = 1/5. (*c*) If growth rate exceeds death rate, the optimal strategy is indicated by the black star labelled L.

We follow previous analyses in evaluating how the optimal reproductive strategy reflects a trade-off between offspring size and offspring number (e.g. [1–3]). In describing the model, we assume that maternal resources each time the adult breeds are fixed and there is an allocation trade-off between number, *N*_0_, and size, *m*_0_, of offspring such that total biomass, *N*_0_ *m*_0_, is constant. Our results depend critically on this specific form of the trade-off. On logarithmic axes, the constraint that *N*_0_ *m*_0_ is constant appears as a straight line with slope = −1, as illustrated by the red lines in figure 2, and the possible maternal strategies are points along these red lines. We ask how the optimal strategy depends on the growth and death rates of juveniles. We choose to work in terms of *per capita* death rates, *d*, and relative growth rates, *g*, which are rates of growth per unit mass. Both *d* and *g* are given per unit time, which will commonly be year^−1^. We assume that juvenile growth and death rates both depend only on juvenile body mass, *m*.

We write expressions in terms of natural logarithms (base *e*), using subscripts S and L to indicate the extreme strategies: either many small or few large offspring. So the smallest possible offspring size is *m*_S_, and the largest possible, if only one is produced, is *m*_L_. For the present discussion, we define juveniles as individuals of any size greater than *m*_S_ but less than *m*_L_. We write *x* = log_*e*_*m* and *y* = log_*e*_*N*. In these terms, the possible maternal strategies are the points *x*_0_, *y*_0_ on the red lines in figure 2.

Consider the fate of *N*_0_ juveniles of initial mass *m*_0_. If their *per capita* death rate when they are of size *x* is d(*x*), then:

The number surviving from size *x*_0_ to *x*, is

2.1*a* $$N=N_{0}\text{exp}(-\int d(x)dt),$$

where the integration runs from the time when the initial size is *x*_0_ to when size is *x*. The relative growth rate, (1/m)(dm/dt), is equal to d*x*/d*t*, let this be written *g*(*x*). Substituting for d*t* in equation (2.1*a*) gives the following

The number surviving from size *x*_0_ to *x*, is

2.1*b* $$N=N_{0}\text{exp}\left(-\overset{x}{\underset{x_{0}}{\int }}\frac{\text{d}(x)}{g(x)}\text{d}x\right),$$

i.e.

2.1*c* $$y=y_{0}-\int_{x_{0}}^{x}\frac{\text{d(}x)}{g(x)}\text{d}x.$$

As the logarithm of the number of offspring surviving to the largest possible size *m*_L_ is our operational measure of fitness, *F* = *y*_L_, we can substitute this and rearrange equation (2.1*c*), to obtain the following:

2.2 $$y_{0}=F+\int_{x_{0}}^{x_{L}}\frac{\text{d}(x)}{g(x)}\text{d}x.$$

**Figure 3.**
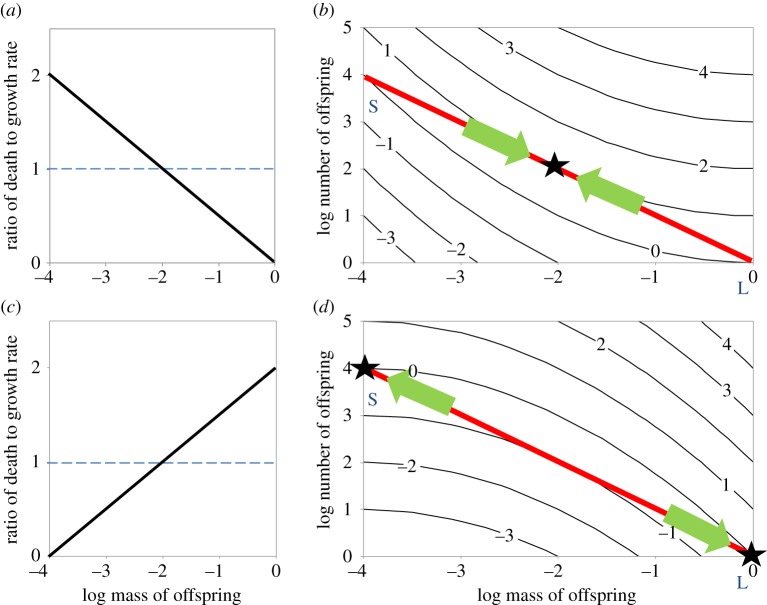
Fitness contours plotted as in figure 2 together with the ratios of death to growth rates that generate them. (*a*,*c*) show the ratio of death to growth rate plotted in relation to offspring mass, dashed horizontal lines show where death rate = growth rate. (*b*,*d*) show the resulting fitness contours. In (*a*,*b*), *d*/*g* = −½ log(offspring mass) and this results in concave fitness contours, selecting for an intermediate optimum. In (*c*,*d*), *d*/*g* = 2 + ½ log(offspring mass) and the resulting fitness contours are convex. This selects for extreme strategies. See text for further details.

The above equation can be used to plot fitness contours, showing the fitness of *x*_0_, *y*_0_ strategies, as in figures 2*b,c* and 3. Selection favours evolutionary change perpendicular to the fitness contours, but the options are constrained to lie on the red trade-off line. Figure 2 shows the conditions when the ratio of the death rate to the growth rate remains constant over offspring size. If the relative growth rate, *g*, is greater than the death rate, *d*, then the smallest size strategy S is fitter, because the fitness contours have shallower slope than the reproductive options (black lines shallower than red line in figure 2*b*). The net result is evolution along the red line towards S. Conversely, if the death rate of juveniles is greater than the growth rate, then the largest size strategy L is fitter, as in figure 2*c*.

If death rate and/or growth rate change with increasing offspring size, then fitness may reach a minimum or maximum at an intermediate position on the constraint line as shown in figure 3. If the death rate shifts from being greater to lower than the growth rate as juvenile size increases, then the fitness contours are concave and optimal strategy is to produce offspring of intermediate size as in figure 3*b*. Conversely, if growth rate is higher than the death rate at the smallest sizes but this reverses as juvenile size increases, then the fitness contours are convex and optimal strategy is to produce offspring of one or the other of the extreme sizes as in figure 3*d*.

## 3. Fitness contours have the same shapes as mass–abundance curves

**Figure 4.**
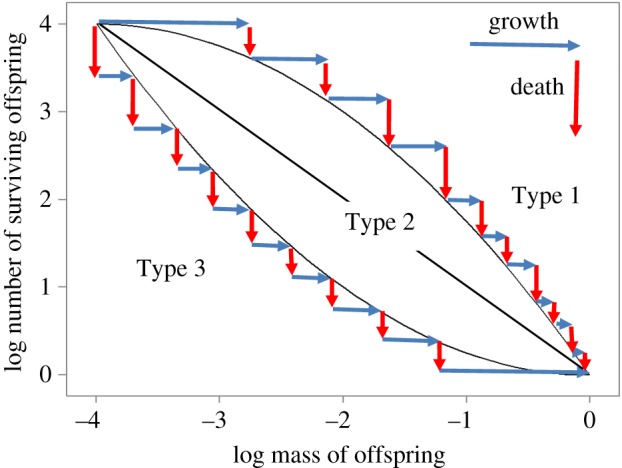
Examples of theoretically possible mass–abundance curves. Initially there are e^4^ juveniles (we use natural logarithms) of mass e^−4^, but as these grow not all survive, and finally for populations in steady state, at most one juvenile remains, of the largest possible size (log mass = 0). Horizontal displacements (blue arrows) indicate growth rates, vertical displacements death rates. Using logarithmic axes scales, the lengths of the arrows indicate relative growth rates and *per capita* death rates. In convex Type 1 curves, the growth rates are initially greater but finally less than the death rates, in concave Type 3 curves the reverse. In log-linear or Type 2 curves, the ratio of death rate to growth rate remains constant over ontogeny.

Until now, we have considered the fitness consequences of the mother producing some combination of number and size of offspring, and we have seen how the optimal strategy depends on the shape of the fitness contours in a space with axes the number and size of offspring. In broad terms, the conclusion is that optimal offspring size depends on how growth and death rates vary as the offspring develops. But, how growth and death rates vary over ontogeny is also key to the characterization of life histories. Life histories have traditionally been described by survivorship and growth curves, plotting the logarithm of survivorship as a function of age, and body mass as a function of age. Pearl [21] plotted survivorship as a function of age and categorized the empirical curves into qualitative ‘types’ based on their shapes. Here, we are concerned only with survivorship and growth of juveniles (i.e. from hatching or birth until the age of first reproduction). The slope of the log survivorship curve is the *per capita* death rate, and the slope of a log body mass curve is the relative growth rate. In the following sections, we will show that because both survivorship and growth curves are functions of age, age can be factored out, allowing us to express the number of surviving offspring as a function of their individual body masses, as in figure 4. This enables us to obtain what we term *mass–abundance curves*. Mass–abundance curves have the same shapes as fitness contours, because they were generated by the same death and growth functions (see the electronic supplementary material for formal proof). So, the convex Type 1 curve in figure 4 has the same shape as the fitness contours in figure 3*d*, and the concave Type 3 curve has the same shape as the contours in figure 3*b*.

## 4. Inferring fitness contours from juvenile growth and survivorship curves

**Figure 5.**
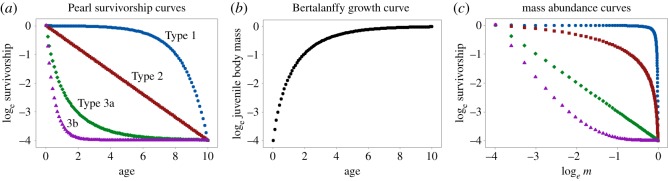
How juvenile survivorship and growth curves as a function of age together determine the shape of mass–abundance curves. Points are plotted each time unit. (*a*) Juvenile survivorship curves of the three types identified by Pearl [21], plotting the logarithm of the proportion of surviving individuals against age. Type 3 is here subdivided into Types 3a and 3b. (*b*) A Bertalanffy growth curve plotting the logarithm of juvenile body mass against age. (*c*) Survivorship and growth curves together determine the shape of mass–abundance curves, and these are identical in shape to fitness contours. Colour coding as in (*a*). The critical log-linear survivorship curve (Type 3a) is the one giving rise to a linear mass–abundance curve.

Mass–abundance curves can be constructed from juvenile survivorship and growth curves in relation to age. This is because the growth curve gives body mass as a function of age, so the inverse function gives age in relation to body mass, and this allows survivorship to be rewritten as a function of body mass. A worked example is given in figure 5. Although our results are general, for realism as well as ease of exposition we assume the growth curves are of Bertalanffy type, an example is shown in figure 5*b*.

Of the types of survivorship curves designated by Pearl ([21]; figure 5*a*), three are relevant to juvenile survivorship considered here: log linear (Pearl Type 2), where the *per capita* death rate is constant, independent of age; convex (Pearl Type 1), where death rate is low initially and increases with age; and concave (Pearl Type 3), where death rate is high initially and decreases with age. Together, growth and survivorship curves determine the shape of mass–abundance curves, as shown in figure 5*c*. Important here is the consideration that the survivorship curves might theoretically be any of the three types (death rate independent of age, high early, or highly late in ontogeny), depending on the causes of mortality in the environment. By contrast, Bertalanffy and other realistic growth curves are constrained by physiology so that growth rate decreases with age and converges to zero as adult size is approached. This is indicated by the spacing between the points representing equal time intervals in figure 5*b*.

To see how the growth and survivorship curves determine the shape of mass–abundance curves, note that the points in figure 5 are plotted at consecutive time intervals. There is a large increase in the logarithm of mass in the first time interval (figure 5*b*) and the first two points in the mass–abundance plot are horizontally displaced by an equivalent distance (figure 5*c*). The decrease in survivorship in the first time interval is small for the blue points (Type 1) but large for the purple points (Type 3b) (figure 5*a*). The vertical displacements in figure 5*c* are the same as those in figure 5*a*. In this way, the displacements in figure 5*a,b* determine the vector displacements in figure 5*c*. The net effects of growth and death rates determine the mass–abundance curves. If growth exceeds death rate, the mass–abundance curve—and the corresponding fitness contour (see previous section)—is shallow. If death exceeds the growth rate, the mass–abundance curve is steep. Where growth and death rates are equal throughout ontogeny, the mass–abundance curve and the corresponding fitness contour are straight lines.

Straight-line fitness contours, corresponding to the straight mass–abundance curves in figure 5*c*, are of special interest because they separate convex from concave contours, and these have opposite selective consequences, as discussed earlier. What determines the shape of the critical straight fitness contour that separates the two selective regimes? Straight fitness contours occur when the survivorship curve (Type 3a in figure 5*a*) is the exact inverse or mirror image of the growth curve (figure 5*b*). This occurs when growth and death rates are equal throughout ontogeny; as relative body mass increases, *per capita* survivorship decreases by exactly the same amount.

## 5. Discussion

Our key results are that: (1) convex fitness contours select for extreme strategies, either many small or few large offspring (figure 3*d*), whereas concave contours select for offspring of intermediate size (figure 3*b*). (2) Straight-line fitness contours as in figure 5*c* are of special interest because they separate convex from concave contours, and from regimes which select for opposite strategies. (3) Fitness contours are identical to *mass–abundance curves* which express the abundance of surviving offspring as a function of their body masses. Mass–abundance curves can be derived from juvenile growth and survivorship curves. (4) The critical straight-line fitness contours occur when survivorship curves are mirror images of growth curves. When growth curves are known or can be assumed theoretically, this allows construction of critical survivorship curves, separating selective regimes with opposite consequences. Our results depend on the assumption of an allocation trade-off such that the product of offspring size and number is fixed, and will need modification if the trade-off takes another form.

To predict the selective consequences for offspring size, we invert the growth curve to generate the critical Type 3a survivorship curve shown in figure 5*a*. If reported survivorship curves are more curved than this, as in Type 3b, then intermediate offspring size is predicted. This is because very small offspring would experience death rates higher than growth rates, and the optimal maternal strategy is to allocate maternal resources to a smaller number of larger offspring. Conversely, if the reported survivorship curve is less curved than the critical Type 3a survivorship curve, or convex as in Type 1, then offspring sizes should be extreme, either as small or as large as possible. So, to predict selective consequences, we need to compare juvenile growth and survivorship curves.

**Figure 6.**
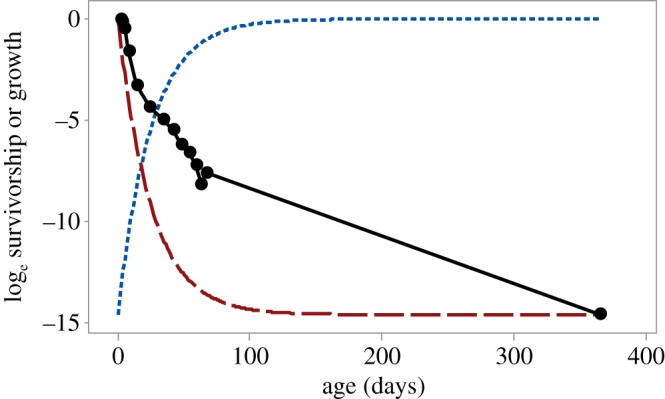
Survivorship and growth curves for mackerel in the NW Atlantic. Survivorship (black solid circles and continuous line) from Ito [22]. Growth curve (blue dotted line) from Simard *et al*. [23], adjusted to steady state so that mass at 1 year equals initial mass/(survivorship at 1 year). The inverted growth curve gives the critical Type 3a survivorship curve and this is shown by the broken red line.

Juvenile growth and survivorship curves are shown in figure 6 for mackerel (*Scomber scombrus*) from egg to first breeding at one year old. The inverted growth curve (broken red line) represents the critical Pearl Type 3a survivorship curve. The real survivorship curve is less concave than the critical curve, meaning that selection favours an extreme strategy, here eggs as small and numerous as possible.

We have shown that the critical Pearl Type 3A survivorship curve has special importance (figure 5). It represents a threshold, separating selection regimes that favour either extreme or intermediate strategies. Its parameters are derived from those of the growth curve, as shown in figure 6. In figure 6, the growth curve is of Gompertz type, but the critical Pearl survivorship curve can always be derived by simply inverting the appropriate growth curve. These very general results hold because mass–abundance curves and juvenile survivorship and growth curves are inextricably linked. If any two are known, the third can be calculated.

Our central thesis is that the key to understanding the tuna reproductive strategy lies in convex fitness contours (figure 3*d*). This is consistent with Olsson *et al.*'s interpretation [3] based on metabolic scaling that relatively higher growth rates early in ontogeny are later replaced by relatively higher death rates due to increased density-dependent mortality as juveniles grow to larger body sizes (see also [24]. Both Olsson *et al.*'s explanation and our simpler, more general theory invoke assumptions that give rise to bowed-outwards mass–survivorship curves and, in our case, to the convex fitness contours that predict two alternative evolutionarily stable extreme strategies. Our theory predicts that for teleosts the ratio of growth to death rates varies systematically as offspring size increases, so *g* > *d* when newborn offspring are very small but *g* < *d* later in ontogeny as juveniles approach mature size.

The theory allows us to offer the following preliminary interpretation of theoretical questions and empirical patterns in the life histories and evolution of teleosts and chondrichthyes:

(1) *Sizes of newborn chondrichthyes.* Why don't sharks produce smaller offspring? Not only are the offspring of sharks and rays about six orders of magnitude heavier than those of hatchling teleosts (figure 1), but also there is a strong positive correlation between the logarithms of body size of newborn and adult chondrichthyes (*r*_96_ = 0.82, p≪0.001, figure 1; [8]). This begs the question of why the largest sharks and rays do not produce larger numbers of smaller offspring than they do—more like tunas. The theory suggests the answer: the protection afforded by large eggs and internal development of sharks are adaptations to reduce mortality of juveniles. High death rates impose strong selection, so it is advantageous for adult chondrichthyes to produce the largest possible offspring consistent with the size–number allocation trade-off. The explanation of the quantitative form of the relationship between offspring size and adult size in chondrichthyes is an open question of great general interest, but beyond the scope of the present paper.
(2) *Sizes of teleost eggs.* Why don't teleosts produce larger offspring? Figure 1 shows that the eggs of marine teleosts are uniformly very small: on average about one milligram (0.001 g), so about 4 orders of magnitude lighter than the smallest newborn sharks and rays [6–8]. However, two lineages of freshwater teleosts, the families Poeciliidae and Zenarchopteridae, have independently evolved life histories with internal fertilization, retention of developing eggs within the mother, and live birth—a suite of traits similar to the chondrichthyes. Given the approximately equal diversity of marine and freshwater teleosts (15 000 versus 13 000 species), it seems that there must be some inherent advantage of producing large numbers of very small, externally fertilized eggs in the marine environment or some powerful constraint against producing large offspring. The question of how small eggs can be is also of interest. We presume that minimum egg size somehow reflects the volume of the zygote required to contain the genetic material, fuel for embryonic development, and related machinery and compounds. The marine teleost zygotes must contain the materials for excreting salts and curtailing water loss in the oceans.
(3) *Exceptional examples.* Additional insights come from the relatively few marine teleosts that lay relatively large eggs and/or provide some degree of parental care. No marine teleost lineages have gone farther than having males protect and sometimes supply nutrition to eggs. The phylogenetically related seahorses and pipefish are examples. Their females produce large numbers of very small eggs, which are fertilized externally before being taken up by the male and brooded and nourished for some substantial period of development [25].
(4) *Historical constraints*. The evolutionary histories of fishes, and more generally of the early vertebrates, suggest important roles for physiological constraints in their phylogenetic, geographical and environmental histories. Reconstructions of the fossil and phylogenetic histories of major lineages suggest that the physiology of osmoregulation, especially in early ontogeny, played a key role in preventing or allowing colonization of freshwater and marine (and ultimately terrestrial) environments.

It appears that all extant lineages of vertebrates probably evolved from ancestors that at one time lived in freshwater environments, as evidenced by internal osmotic concentrations more dilute than seawater. The ancestors of chondrichthyes and coelacanths were colonized from fresh water and diversified in the oceans in the late Ordovician to mid-Devonian (approx. 400—395 Ma, respectively; [26,27]). The approximately 1000 extant species of chimeras, sharks and rays share with the ‘living fossil’ coelacanth life histories based on internal fertilization and production of a few large well-developed offspring, born or hatched with organs capable of maintaining osmotic balance [28–32].

By contrast, the teleosts invaded the oceans from fresh waters much more recently, probably in the Jurassic (approx. 200 Ma; [33,34]). They diversified explosively after the Cretaceous-Tertiary mass extinction event (65 Ma) had eliminated several of lineages of chondrichthyes and invertebrates. With approximately 18 000 extant species, teleosts are by far the most diverse group of marine vertebrates. Although some of their success is probably due to jaw structure and other morphological innovations, much of it must be attributed to their diverse body sizes, which span more than 8 orders of magnitude (0.005–2 000 000 g) and allow them to exploit an enormous variety of niches. The diversification of marine teleosts was facilitated by their ability to produce tiny eggs that are externally fertilized, maintain osmotic balance as the embryos develop in seawater, and hatch to give rise to fully functional offspring able to feed themselves and survive independently of their parents [35–38]. There is abundant room to explore further the detailed relationships between fossil and phylogenetic history, osmoregulatory physiology and life history which are only hinted at here.

We conclude that the life histories of marine chondrichthyes and teleosts represent adaptive responses to the fundamental constraint of the number–size allocation trade-off. The two extreme strategies are maintained because the alternative few-large and many-small strategies represent two peaks of high fitness separated by a valley of low fitness representing moderate numbers of offspring of intermediate sizes. Retaining a few large eggs or embryos within the mother allows sharks to recruit new adults because the slow-growing offspring are protected from high rates of mortality. Producing millions of miniscule eggs allows tunas to recruit new adults because some of the newly hatched larvae grow fast enough to survive to adulthood, despite high mortality due to competition and predation in the plankton.

## Supplementary Material

Data of Fig. 1

## Supplementary Material

Proof that mass-abundance curves have the same shapes as fitness contours

## Data accessibility

The data of figure 1 are available in the electronic supplementary material.

## References

[1] Charnov EL, Ernest SKM 2006 The offspring-size/clutch-size trade-off in mammals. Am. Nat. 167, 578–582. 16670999 10.1086/501141

[2] Smith CC, Fretwell SD 1974 Optimal balance between size and number of offspring. Am. Nat. 108, 499–506. (10.1086/282929)

[3] Olsson KH, Gislason H, Andersen KH 2016 Differences in density-dependence drive dual offspring size strategies in fish. J. Theor. Biol. 407, 118–127. (10.1016/j.jtbi.2016.07.027) 27457096

[4] Froese R, Pauly D 2016 FishBase. See www.fishbase.org.

[5] Winemiller KO, Rose KA 1992 Patterns of life-history diversification in North American fishes: implications for population regulation. Can. J. Fish. Aquat. Sci. 49, 2196–2218. (10.1139/f92-242)

[6] Winemiller KO, Rose KA 1993 Why do most fish produce so many tiny offspring. Am. Nat. 142, 585–603. (10.1086/285559) 19425962

[7] Merrett NR 1994 Reproduction in the North Atlantic oceanic ichthyofauna and the relationship between fecundity and species sizes. Environ. Biol. Fishes 41, 207–245. (10.1007/bf00023814)

[8] Andersen KH et al. 2016 Characteristic sizes of life in the oceans, from bacteria to whales. Annu. Rev. Mar. Sci. 8, 217–241. (10.1146/annurev-marine-122414-034144) 26163011

[9] Andersen KH, Beyer JE, Pedersen M, Andersen NG, Gislason H 2008 Life-history constraints on the success of the many small eggs reproductive strategy. Theor. Popul. Biol. 73, 490–497. (10.1016/j.tpb.2008.02.001) 18367223

[10] Duarte CM, Alcaraz M 1989 To produce many small or few large eggs: a size-independent reproductive tactic of fish. Oecologia 80, 401–404. (10.1007/bf00379043) 28312069

[11] Elgar MA 1990 Evolutionary compromise between a few large and many small eggs: comparative evidence in teleost fish. Oikos 59, 283–287. (10.2307/3545546)

[12] Neuheimer AB, Hartvig M, Heuschele J, Hylander S, Kiorboe T, Olsson KH, Sainmont J, Andersen KH 2015 Adult and offspring size in the ocean over 17 orders of magnitude follows two life history strategies. Ecology 96, 3303–3311. (10.1890/14-2491.1) 26909435

[13] Freedman JA, Noakes DLG 2002 Why are there no really big bony fishes? A point-of-view on maximum body size in teleosts and elasmobranchs. Rev. Fish Biol. Fish. 12, 403–416. (10.1023/a:1025365210414)

[14] Goodwin NB, Dulvy NK, Reynolds JD 2002 Life-history correlates of the evolution of live bearing in fishes. Phil. Trans. R. Soc. Lond. B 357, 259–267. (10.1098/rstb.2001.0958) 11958695 PMC1692945

[15] Jorgensen C, Auer SK, Reznick DN 2011 A model for optimal offspring size in fish, including live-bearing and parental effects. Am. Nat. 177, E119-E135. (10.1086/659622) 21508600

[16] Shine R 1978 Propagule size and parental care: safe-harbor hypothesis. J. Theor. Biol. 75, 417–424. (10.1016/0022-5193(78)90353-3) 570622

[17] Kiflawi M 2006 On optimal propagule size and developmental time. Oikos 113, 168–173. (10.1111/j.0030-1299.2001.14378.x)

[18] Charnov EL 1997 Trade-off-invariant rules for evolutionarily stable life histories. Nature 387, 393–394. (10.1038/387393a0) 9163423

[19] Charnov EL 1993 Life history invariants: some explorations of symmetry in evolutionary ecology, p. 167 Oxford, UK: Oxford University Press.

[20] Sibly R, Calow P 1987 Ecological compensation: a complication for testing life-history theory. J. Theor. Biol. 125, 177–186. (10.1016/S0022-5193(87)80039-5) 3657207

[21] Pearl R 1928 The rate of living. New York, NY: Knopf.

[22] Ito Y 1980 Comparative ecology, p. 436 Cambridge, UK: Cambridge University Press.

[23] Simard P, Castonguay M, Damours D, Magnan P 1992 Growth comparison between juvenile Atlantic mackerel (*scomber-scombrus*) from the 2 spawning groups of the northwest Atlantic. Can. J. Fish. Aquat. Sci. 49, 2242–2248. (10.1139/f92-245)

[24] Falster DS, Moles AT, Westoby M 2008 A general model for the scaling of offspring size and adult size. Am. Nat. 172, 299–317. (10.1086/589889) 18631112

[25] Wilson AB, Ahnesjo I, Vincent ACJ, Meyer A 2003 The dynamics of male brooding, mating patterns, and sex roles in pipefishes and seahorses (family Syngnathidae). Evolution 57, 1374–1386. (10.1111/j.0014-3820.2003.tb00345.x) 12894945

[26] Benton MJ 2005 Vertebrate palaeontology, 3rd edn Somerset, NJ: Wiley-Blackwell.

[27] Miller RF, Cloutier R, Turner S 2003 The oldest articulated chondrichthyan from the Early Devonian period. Nature 425, 501–504. (10.1038/nature02001) 14523444

[28] Wourms JP 1977 Reproduction and development in chondrichthyan fishes. Am. Zool. 17, 379–410. (10.1093/icb/17.2.379)

[29] Wourms JP, Demski LS 1993 The reproduction and development of sharks, skates, rays and ratfishes: introduction, history, overview, and future prospects. Environ. Biol. Fishes 38, 7–21. (10.1007/bf00842899)

[30] Long JA, Trinajstic K, Young GC, Senden T 2008 Live birth in the Devonian period. Nature 453, 650-U656. (10.1038/nature06966) 18509443

[31] Long JA et al*.* 2015 Copulation in antiarch placoderms and the origin of gnathostome internal fertilization. Nature 517, 196 (10.1038/nature13825) 25327249

[32] Ahlberg P, Trinajstic K, Johanson Z, Long J 2009 Pelvic claspers confirm chondrichthyan-like internal fertilization in arthrodires. Nature 460, 888–889. (10.1038/nature08176) 19597477

[33] Arratia G 2000 Remarkable teleostean fishes from the Late Jurassic of southern Germany and their phylogenetic relationships. Fossil Record 3, 137–179. (10.5194/fr-3-137-2000)

[34] Arratia G, Thies D 2001 A new teleost (*Osteichthyes. Actinopterygii*) from the Early Jurassic Posidonia shale of northern Germany. Fossil Record 4, 167–187. (10.5194/fr-4-167-2001)

[35] Evans DH 2008 Teleost fish osmoregulation: what have we learned since August Krogh, Homer Smith, and Ancel Keys. Am. J. Physiol.: Regul. Integr. Comp. Physiol. 295, R704–R713. (10.1152/ajpregu.90337.2008) 18525009

[36] Alderdice DF 1988 Osmotic and ionic regulation in teleost eggs and larvae. In Fish physiology (eds Hoar WS, Randall DJ), pp. 163–251. San Diego, CA: Academic Press.

[37] Fyhn HJ, Finn RN, Reith M, Norberg B 1999 Yolk protein hydrolysis and oocyte free amino acids as key features in the adaptive evolution of teleost fishes to seawater. Sarsia 84, 451–456. (10.1080/00364827.1999.10807350)

[38] Varsamos S, Nebel C, Charmantier G 2005 Ontogeny of osmoregulation in postembryonic fish: a review. Comp. Biochem. Physiol. A: Mol. Integr. Physiol. 141, 401–429. (10.1016/j.cbpb.2005.01.013) 16140237

