# Supplementary material for: The shark-tuna dichotomy: why tuna lay tiny eggs but sharks produce large offspring
Source: R Soc Open Sci. 2018 Aug 15;5(8):180453. doi: 10.1098/rsos.180453 (PMC6124039; doi:10.1098/rsos.180453)
Supplement: Proof that mass-abundance curves have the same shapes as fitness contours [file rsos180453supp2.docx]

**Fitness contours have the same shape as the corresponding mass-abundance curves.**

Here we show that the shape of the fitness contours is the same as the shape of the corresponding mass-abundance curves. So the Type 1 curve in Fig. 4 has the same shape as the fitness contours in Fig. 3d, and the Type 3 curve has the same shape as the contours in Fig. 3b. The reason is that these curves were generated by the same death and growth functions, with the ratios shown in Fig. 4a and c. To see why fitness contours necessarily have the same shape as corresponding mass-abundance curves, note that from equation 1c, a mass abundance curve starting at S has equation

y(x) = y_S_ $-\int_{x_{S}}^{x} \frac{d\left( x \right)}{g\left( x \right)}dx,$ (A1)

and writing h(x) = d(x)/g(x), from equation 2 fitness contours have equation

y(x) = F + $\int_{x}^{x_{L}} h(x)dx$, (A2)

where we have replaced x_0_ and y_0_ by x and y respectively because we are now thinking of the contours as plotted in (x, y) space.

Writing H(z) for the integral of h(x) evaluated at x=z, the equation of the mass-abundance curves and the fitness contours are

y(x) = y_S_ – H(x) + H(x_S_) (A3)

and

y(x) = F + H(x_L_) – H(x) (A4)

from equations (A1) and (A2) respectively. In the above equations, y_s_, H(x_S_), F and H(x_L_) are constants, the only term that varies with x is H(x), and this dictates the shapes of the curves. Hence fitness contours necessarily have the same shape as corresponding mass-abundance curves if they are generated by the same death and growth functions.
